# Cough reflex and oral chemesthesis induced by capsaicin and capsiate in healthy never-smokers

**DOI:** 10.1186/1745-9974-3-9

**Published:** 2007-10-31

**Authors:** Miyako Yamasaki, Satoru Ebihara, Takae Ebihara, Shannon Freeman, Shinsuke Yamanda, Masanori Asada, Motoki Yoshida, Hiroyuki Arai

**Affiliations:** 1 the Department of Geriatrics and Gerontology, Tohoku University School of Medicine, Seiryo-cho 1-1, Aoba-ku, Sendai, 980-8574, Japan

## Abstract

**Background:**

Many tussive agents are components of foods, but little is known about the relationship between cough reflex and oral chemesthesis sensitivities. We investigated the relationships between cough reflex and oral chemesthesis in individuals using two transient receptor potential vanilloid 1 (TRPV1) agonists with different potencies: capsaicin and capsiate.

**Methods:**

Twenty-eight healthy never-smokers were allocated to evaluate cough and oral chemesthesis of capsinoids. Cough reflex sensitivities are estimated by the lowest concentrations generating five coughs by each TRPV1 agonist inhalation. Oral chemesthesis sensitivities are estimated by the lowest concentrations which generate a hot sensation when filter paper loaded with each TRPV1 agonist is placed on the tongue.

**Results:**

There were strong correlations between capsaicin- and capsiate-induced cough reflex sensitivities, and between capsaicin- and capsiate-induced oral chemesthesis sensitivities. However, there were no significant correlations between cough reflex and oral chemesthesis sensitivities induced by both capsaicin and capsiate. The cough reflex sensitivities are significantly greater in females than in males whereas there were no gender differences in oral chemesthesis.

**Conclusion:**

The results showed that the sensitivities of sensory afferents were different between cough reflex and oral chemesthesis, suggesting that TRPV1 sensitivities differ between organs within healthy individuals. Capsiate could be a tussigen for the cough challenge test.

## Background

Although many tussive agents, such as capsaicin, citric acid, and acetic acid, are components of foods, it is unknown whether these chemical stimuli equally stimulate sensory nerves in bronchial airways and the oral cavity. The inhalation of tussive agents as a cough challenge test is a useful method to quantify cough in a clinical setting and to assess the antitussive effects of specific therapies in a laboratory setting [1]. The inhalation cough challenge is applied via the oral cavity, but little attention has been paid to the effects of tussive agents on oral sensory systems during the cough challenge test. Although, while testing and developing the inhalation cough challenges, a large number of tussive agents have been tried, capsaicin has stood the test of time and nowadays is the most widely used probably as a result of greater reproducibility and safety [1]. In contrast to classical tastes such as sweet, salty, bitter, sour and umami, the oral sensation induced by capsaicin is called chemesthesis, a sensation of irritation produced by chemical stimulation and mediated by the trigeminal nerve [2].

The physiological effects of capsaicin on cough may be modulated by oral sensory stimuli. Activation of capsaicin-sensitive afferents in the tongue and palate evoke local release of neuropeptides such as substance P and calcitonin gene-related peptides, which are contained in the nerve terminal of the sensory neurons [3,4]. The neuropeptides exert powerful vasoactive and secretomoter effects leading to vasodilation, plasma exudation, triggering reflex salivation and an increase in the secretion of mucus in the airway. Capsaicin is a potent gustatory stimulus which may also promote airway secretions. Gustatory rhinorrhoea has been shown to occur after eating spicy foods and this observation demonstrates a link between gustation and airway secretion of mucus [5]. There is also a possibility that capsaicin in the oral cavity induces bronchoconstriction the same as intranasal application of capsaicin elicits bronchoconstriction [6].

Moreover, in the brain, the gustatory fibers and the sensory fibers that initiate cough may interact with each other because of the close anatomical relationship [7]. In order to inquire into the possible modulation of cough reflex by capsinoid-induced oral stimuli, it might be important to know whether there is a relationship between cough reflex and oral sensitivities to capsinoids. In addition, for the same purpose, it may also be important to know whether there is a gender difference in oral sensitivities to capsinoids since cough reflex sensitivity to capsaicin shows prominent gender differences [8,9].

Capsaicin acts mainly on the afferent neurons of the non-myelinated C-fibers by the opening of a non-selective cation channel of capsaicin receptor, transient receptor potential vanilloid 1 (TRPV1) [10]. Capsiate is obtained from faint-pungent cultivar of red peppers named CH-19 Sweet [11]. CH-19 Sweet is a fixed cultivar that was selected and cultivated from a pungent cultivar, CH-19, of pepper. Capsiate is known to activate TRPV1 [12], and, despite faint-pungency, increases adrenaline secretion and oxygen consumption like capsaicin [13]. *Capsium *fruits are used worldwide in foods for their pungency. The pungency felt when eating *Capsium *fruits is mainly attributed to the activation of oral TRPV1 [14].

TRPV1 receptors found on sensory airway nerves are important in the cough reflex [15]. Isolated pulmonary vagal afferent nerves are responsive to TRPV1 stimulation. When one eats foods containing capsaicin, the burning sensation is elicited by TRPV1-containing peptidergic nociceptors surrounding taste buds in the tongue [16].

Capsaicin-induced cough may not solely be mediated through the nerves expressing TRPV1 receptors. Capsaicin inhalation elicits cough through the activation of rapidly adapting receptors (PAR) [17,18]. The activation of PAR is presumably secondary to airway smooth muscle contraction, mucous secretion or edema formation by capsaicin [18]. Therefore, cough induced by capsaicin is a mixture of direct and indirect responses to the capsaicin. The same situations are also proposed for oral chemesthesis. Despite the complexities of the neural network and involved mechanisms to induce cough or oral chemesthesis, the outcome measurements are relatively simple in these phenomena.

In order to investigate the possible relationship between the perception of sensations mediated by TRPV1, whether directly or indirectly, in different organs, e.g. lung and tongue within individuals, we compared cough reflex and oral chemesthesis sensitivities using two TRPV1 agonists with differential potencies, capsaicin and capsiate. In addition, we evaluated the possibility of the use of capsiate as a cough challenge test.

## Methods

### Subjects and protocols

Twenty-eight healthy never-smokers (14 male, 14 female) were allocated to evaluate cough and oral chemesthesis of capsinoids. All were originally recruited via public postings in and around the Tohoku University School of Medicine campus. The mean age was 36.4 ± 2.3 (SE) years. The study was approved by the Institutional Review Boards of Tohoku University School of Medicine. Subjects were without history of pulmonary disease, recent (within 4 weeks) suggestive symptoms, respiratory tract infection and seasonal allergies. Subjects did not take any regular medication.

Subjects underwent the sensitivity tests on four successive days at 10:00 am. Each of the four days was assigned to the capsaicin cough sensitivity test, the capsaicin oral chemethesis test, the capsiate cough sensitivity test, or the capsiate oral chemesthesis test. The order of the four tests was randomly decided using a computer program. The day before the start of the test and during the four days, subjects were prohibited from taking any capsinoids in meals or beverages. In order to ensure subjects avoid consumption of capsinoids during meals, various foods and dishes that contain them were explained to the subjects.

### Cough reflex sensivity tests for capsaicin and capsiate

Cough reflex sensitivities to capsaicin and capsiate were measured on different days using the modification of the method by Fujimura and colleagues [8]. 30.5 mg of Capsaicin (Sigma Aldrich, Seatle, USA) was dissolved in Tween 80 (1 ml) and ethanol (1 ml) and then dissolved in physiological saline (8 ml) to make a stock solution of 0.01 M, which was stored at -20°C. This solution was diluted with physiological saline to make testing solutions starting at a concentration of 0.49 μM and increasing it by doubling the concentration up to 1000 μM.

**Figure 1:**
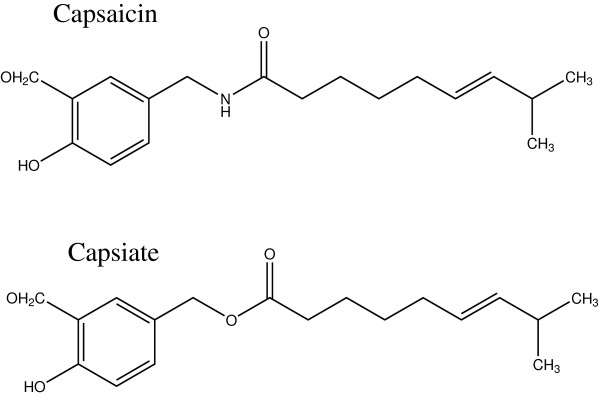
Structures of capsaicin and capsiate.

Capsiate was extracted from CH-19 sweet (kind gift from Ajinomoto KK, Kawasaki, Japan). Compared with capsaicin, capsiate has an ester bond instead of the amide bond between the vanillyl moiety and fatty acid chain (Figure 1). Harvested chili peppers (CH-19 sweet) were washed and dried. Then the crude oil was extracted from the dried chili peppers using n-hexane. The crude oil was refined by the distillation and the column chromatography. Finally, in order to adjust the concentration, the refined oil was diluted with medium-chain triglyceride. In this original capsiate extract solution, the capsiate content of the sample was ~7%. The rest of the extract solution was mainly caprylic acid. Capsaicin was less than 0.0001% among capsinoids. 70 μl of capsiate extract was dissolved in Tween 80 (1 ml) and ethanol (1 ml), and then dissolved in physiological saline (19 ml) to make a solution of 0.01 M. This solution was diluted with physiological saline to make testing solutions starting at a concentration of 0.49 μM and increasing it by doubling the concentration up to 1000 μM. Capsiate was diluted from the original extract solution every time just before the sensitivity test.

Each subject inhaled a control solution of physiological saline followed by a progressively increasing concentration of capsaicin or capsiate solution. Solutions were inhaled for 15 s every 60 s, by tidal mouth-breathing, while wearing a nose-clip from a Bennett twin nebulizer (3012-60cc; Puritam-Bennett Co., Carsbad, CA, USA). Increasing concentrations were inhaled until five or more coughs were elicited. The nebulizer output was 0.21 ml/min. The cough reflex sensitivities to capsaicin and capsiate were defined as the lowest concentration of capsaicin or capsiate that elicited five or more coughs (C5). In our preliminary experiments, it was confirmed that the Tween 80 and/or caprylic acid dilutions at any concentration used in saline without capsinoids did not induce cough for 15 s inhalation.

### Oral chemesthesis measurements

Chemesthesis to capsaicin and capsiate was measured with a modification of the semi-quantitative clinical gustometry using a filter-paper disc, which is routinely used for the evaluation of dysgeusia in a clinical setting [19]. Again, chemesthesis to capsaicin and capsiate were measured on different days. The testing solutions were prepared for both capsaicin and capsiate in the same way as the cough reflex sensitivity measurements, but distilled water was used instead of physiological saline. A droplet of each testing solution was added to the filter paper disc (8 mm diameter), and then the disc was placed on the left side of the tongue 2 cm from the tip (i.e. locus for left cholda tympani nerve), for one second. The filter discs with the progressively increasing concentrations of capsaicin or capsiate were applied every 5 min, and the subject was asked to gargle with distilled water during the interval. Because irritant sensations take longer than classical tastes, subjects were instructed to wait 10 s before making a conclusion on their chemesthesis [16]. The chemesthesis to capsaicin and capsiate were defined as the lowest concentration of capsaicin or capsiate that elicited a pungent or burning sensation for the subject. Although capsinoids have the possibility to elicit bitterness, the subject was asked to ignore the bitterness [20].

In our preliminary experiments, it was confirmed that the Tween 80 and/or caprylic acid dilutions at any comparable concentrations in distilled water without capsinoids did not induce oral chenesthesis, and it was certified that there was no tachyphylaxis of responses to capsinoids with 24-hour intervals for both cough reflex sensitivities oral chemesthesis.

### Statistical analysis

Results are expressexd as mean ± SE. Comparisons between each threshold concentration in differential stimuli were performed by a paired t-test. Comparisons between the sensitivities in males and females were performed by the Mann-Whitney test. The correlations between each threshold concentration in differential stimuli were estimated by Pearson's correlation coefficient. A value of p < 0.05 was considered statistically significant.

## Results

Both cough reflex sensitivities and oral chemesthesis tests were performed without any unpleasant feelings or side effects after the tests for all subjects. The mean threshold concentration to induce cough (log C_5 _value) was significantly greater in capsiate (2.55 ± 0.09 log μM) than in capsaicin (1.20 ± 0.09 log μM) (p < 0.0001). The mean threshold concentration to induce oral chemesthesis by capsiate (2.22 ± 0.10 log μM) was significantly greater than that by capsaicin (1.55 ± 0.11 log μM) (p < 0.0001). The mean threshold concentration for capsaicin application was significantly greater in cough reflex sensitivity than that in oral chemesthesis (p < 0.03).

The mean threshold concentration for capsiate application was significantly greater in cough reflex sensitivity than in oral chemesthesis (p < 0.01).

**Figure 2:**
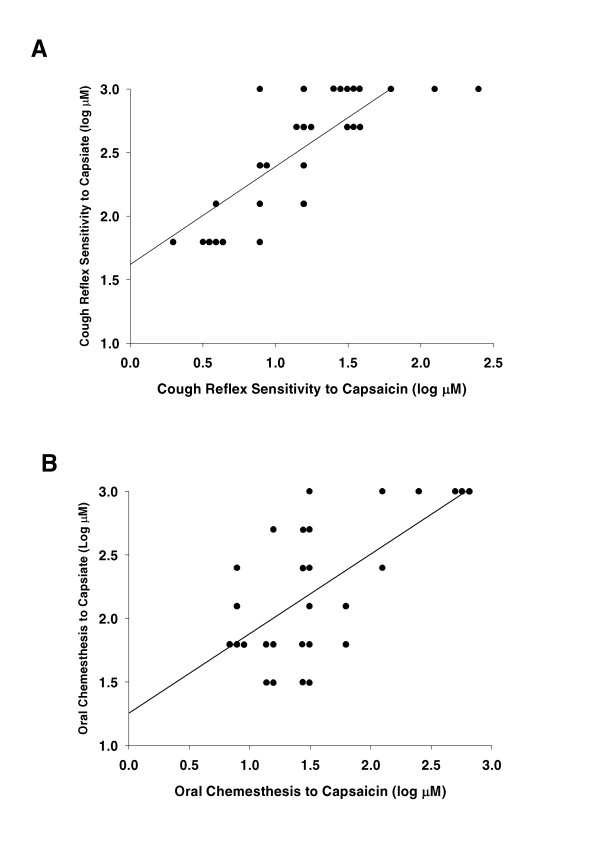
Correlations between capsaicin- and capsiate-induced cough reflex sensitivities (A), and between capsaicin- and capsiate-induced oral chemesthesis sensitivities (B). The solid lines represent regression lines.

As shown in Figure 2A, there was a strong correlation between capsaicin- and capsiate-induced cough reflex sensitivities (r = 0.79, p < 0.001). Similarly, as shown in Figure 2B, there was a strong correlation between capsaicin- and capsiate-induced oral chemesthesis sensitivities (r = 0.64, p < 0.01). These results suggest that cough reflex and pungent sensation are induced by stimulation of TRPV1 in each responsible organ.

However, there was no significant correlation between cough reflex and pungent taste sensitivities induced by capsaicin (r = -0.12, p = 0.50). Similarly, there was no significant correlation between cough reflex and pungent taste sensitivities induced by capsiate (r = 0.30, p = 0.22). These results suggest that the same TRPV1 stimulation induce differential strength of sensation according to the organs within individuals.

Table 1 shows cough reflex sensitivities and oral chemesthesis classified by gender. The threshold concentrations to induce cough reflex are significantly greater in males than those in females for both capsaicin and capsiate (p < 0.03 and p < 0.05, respectively). However, in oral chemesthesis, there were no significant differences between males and females for both capsaicin and capsiate.

**Table 1:** Gender differences in cough reflex sensitivities and oral chemesthesis

	Male	Female	p value
Number	14	14	
Age (year)	34.2 ± 2.0	38.5 ± 4.1	n.s.
Cough reflex sensitivity			
Capsaicin (Log μM)	1.41 ± 0.12	1.00 ± 0.11	<0.03
Capsiate (Log μM)	2.72 ± 0.10	2.37 ± 0.13	<0.05
Oral chemesthesis			
Capsaicin (Log μM)	1.51 ± 0.17	1.58 ± 0.13	n.s.
Capsiate (Log μM)	2.22 ± 0.15	2.22 ± 0.14	n.s.

Data are mean ± S.E. P-values are comparisons between males and females in each variable by the Mann-Whitney test. n.s. denotes not significant.

## Discussion

In this study, no significant relationship between cough reflex sensitivity and oral chemesthesis to capsinoids within individuals was found. The cough reflex to TRPV1 stimulations are less sensitive in males than in females whereas there was no significant gender difference in the oral chemesthesis to capsinoids. Here we showed that the usefulness of capsinoids with respect to both their action as a tussigen and the capability to evoke oral chemesthesis.

A strong correlation between the threshold concentrations between capsaicin- and capsiate-induced cough was found. Similarly, the threshold concentrations between capsaicin- and capsiate-induced oral chemesthesis significantly correlated. In both sensations, capsiate required a much higher concentration than capsaicin. The intragastric administration of capsiate increases adrenalin secretion and oxygen consumption in mice [21,22]. In addition, capsiate suppresses T cell activation by inhibiting NF-κB-dependent transcriptional activity [23]. These studies suggest that capsiate shares biological activities with capsaicin in spite of very weak pungency. However, the reasons for the weak pungency of capsiate are not clear. Iida and colleagues speculated that less accessibility of capsiate to nociceptors due to its lipophilicity might contribute to the weak pungency [12]. In our studies, the difference in threshold concentration between capsiate and capsaicin are greater in cough reflex sensitivity than oral chemesthesis. This may reflect lower accessibility to TRPV1 responsible for cough reflex than that for oral chemesthesis.

Individual variations in cough reflex sensitivities were shown in the cough challenge test even in healthy subjects. The variation exists regardless of methods of cough challenge and tussive stimulants. Cough reflex is reportedly less sensitive in men than women [8,9]. Although oral chemesthesis also exhibits variability, a gender difference has not been investigated as far as we know. In our study, the gender difference in cough reflex sensitivities is consistent with previous observations, suggesting methodological appropriateness even with capsiate. We observed no gender difference in oral chemesthesis in healthy subjects using two TRPV1 agonists with different potencies. There are several reports showing an association between oral chemesthesis and taste perception [24,25]. However, the results of the gender difference in taste perceptions are conflicting according to the stimuli and methods [26]. Nasal chemesthesis is relatively better investigated than oral chemesthesis because nasal irritation is an important issue in environmental public health, and data about gender differences are conflicting [27]. In contrast to chemesthesis, gender dependency in pain perception is well documented [28]. Numerous studies demonstrated that certain pain disorders occur with higher prevalence, intensity, or duration in women than in men [29].

The explanation for an increase in cough reflex sensitivity in healthy females is unknown. One hypothesis is an endocrine influence on the cough reflex. Recently, prolactin was reported to enhance TRPV1 response in the presence of estrogen in rat sensory neurons [30]. However, previous studies showing that postmenopausal women have greater cough reflex sensitivity than premenopausal women [8], and more frequently suffer from angiotensin-converting enzyme inhibitor-induced cough [31] would argue against this hypothesis. In addition, our result showing no gender difference in oral chemesthesis may also conflict with the systemic influence of sex hormones on gender differences.

Both the peripheral and central explanations for why oral chemesthesis are not correlated to cough reflex sensitivity are postulated. The lack of relationship between oral chemesthesis and cough reflex sensitivity within individuals may suggest a differential expression of TRPV1 according to the organs within individuals. In patients with chronic cough, increased expression of TRPV1 in airway nerves was reported [15]. Inflammatory bowel disease is associated with the upregulation of TRPV1 in the nerve fibers of the colon [32]. Taste performance on the human tongue varies with the density of fungiform taste buds, which are heavily innervated by chemesthesis receptor neurons [33]. Thus, the organ specific up-regulation of TRPV1 is found in diseases. Differential oral chemesthesis could result from the differential number of TRPV1 in the tongue.

More importantly, the differential sensitivities to capsinoids between cough reflex and oral chemesthesis could be reflected in the differential contribution of indirect activation of afferent neurons. In cough response, capsaicin is known to activate not only C-fibers that have TRPV1 but also rapidly adapting airway mechanoreceptors (PAR) that do not have TRPV1 [17,18]. PAR is activated by a large number of mechanical and chemical irritant stimuli, by inflammatory and immunological mediators, and by airway and lung pathological changes [34]. Presumably, capsaicin activates PAR indirectly by contraction of airway smooth muscle or by an increase in extracellular liquid, or by both mechanisms [34]. Thus, the secondary effect of capsaicin is not small on cough reflex sensitivities. On the other hand, indirect effects of capsaicin on oral chemesethesis sensations have not yet been identified, suggesting that the indirect effect might be negligible in oral chemesthesis.

Besides the peripheral factors, central factors may be involved in the differential sensitivities of TRPV1 stimulation between cough reflex and oral chemesthesis within individuals. In contrast to oral chemesthesis, which was finally integrated by cortical processing, cough reflex is essentially a brainstem reflex. Therefore, there is a possibility that the gain of a cortical neural process is involved in the differences in oral chemesthesis, but not in cough reflex. Evidence of gustatory brainstem taste nuclei and cortical connections, which potentially modulate these processes, provide a plausible neural basis for a central gain mechanism [35,36]. Recently, the possible modification of cough reflex by the brain cortex was highlighted [37,38]. There are several studies as to the functions of supramedullary areas responsible for cough. The interaction between sweet taste stimulation and cough reflex was suggested [39]. If the urge-to-cough which precedes coughing was measured, we could more easily understand the lack of relationship between oral chemesthesis and cough reflex sensitivity [40]. Further studies are required to elucidate the relationships between cough reflex and sensory inputs to the cortex.

The lack of relationship between oral chemesthesis and cough reflex sensitivity within individuals might suggest the low possibility of a modulatory effect of capsinoids which were deposited in the oral cavity during the cough challenge test. Although the concentration to induce oral chemesthesis to capsinoids is relatively smaller than that of cough reflex, oral chemesthesis did not trigger cough responses in the present healthy subjects. The lack of gender difference in oral chemesthesis also supports the no modulation hypothesis.

In the present study, we found that the capsiate does not induce the sustained irritant airway feeling that is frequently observed in the case of the capsaicin cough challenge test. This might be attributed to the lipohilicity and instability of capsiate. Although this biophysical feature of capsiate is a disadvantage for the preparation procedure, this could be a benefit for the subject to avoid uncomfortable feelings after the cough challenge test [12].

## Conclusion

In conclusion, the results showed that the sensitivities of sensory afferents were different between cough reflex and oral chemesthesis, suggesting that TRPV1 sensitivities differ among organs within healthy individuals. The results also suggest that capsiate could be a useful tussigen for the cough challenge test.

## Competing interests

The author(s) declare that they have no competing interests.

## Authors' contributions

MY, SE and TE participated the design of the study, collected and analyzed data, and drafted the manuscript. SF, SY, AM and MY participated in the design of the study and collected the data. HA participated in design of the study and helped to draft the manuscript. All the authors read and approved the final manuscript.
